# Prefrontal responses to Stroop tasks in subjects with post-traumatic stress disorder assessed by functional near infrared spectroscopy

**DOI:** 10.1038/srep30157

**Published:** 2016-07-25

**Authors:** Amarnath Yennu, Fenghua Tian, Alexa Smith-Osborne, Robert J. Gatchel, Fu Lye Woon, Hanli Liu

**Affiliations:** 1 The University of Texas at Arlington, Department of Bioengineering, Arlington, TX 76019, USA; 2 The University of Texas at Arlington, School of Social Work, Arlington, TX 76019, USA; 3 The University of Texas at Arlington, Department of Psychology, Arlington, TX 76019, USA; 4 The University of Texas Southwestern Medical Center, Dallas, TX 75390, USA

## Abstract

Studies on posttraumatic stress disorder (PTSD) showing attentional deficits have implicated abnormal activities in the frontal lobe. In this study, we utilized multichannel functional near-infrared spectroscopy (fNIRS) to investigate selective attention-related hemodynamic activity in the prefrontal cortex among 15 combat-exposed war-zone veterans with PTSD and 13 age- and gender-matched healthy controls. While performing the incongruent Stroop task, healthy controls showed significant activations in the left lateral prefrontal cortex (LPFC) compared to baseline readings. This observation is consistent with previously reported results. In comparison, subjects with PTSD failed to activate left LPFC during the same Stroop task. Our observations may implicate that subjects with PTSD experienced difficulty in overcoming Stroop interference. We also observed significant negative correlation between task reaction times and hemodynamic responses from left LPFC during the incongruent Stroop task in the PTSD group. Regarding the methodology used in this study, we have learned that an appropriate design of Stroop paradigms is important for meeting an optimal cognitive load which can lead to better brain image contrasts in response to Stroop interference between healthy versus PTSD subjects. Overall, the feasibility of fNIRS for studying and mapping neural correlates of selective attention and interference in subjects with PTSD is reported.

According to American Psychiatric Association, post-traumatic stress disorder (PTSD) is an anxiety disorder that can be developed after exposure to traumatic events, such as combat environment, sexual assault, or the serious injury, resulting in psychological trauma. Patients with PTSD often re-experience the traumatic event in the form of nightmares, intrusive recollections, flashbacks, and physiological arousal and distress in response to reminders of trauma. Hyperarousal symptoms, such as hypervigilance, exaggerated startle, and difficulty sleeping or concentrating are also reported among PTSD patients. The estimated lifetime prevalence of PTSD among American adults is 7.8%^1^. Combat-related PTSD is found in 9–25% of war-zone veterans and is often persistent and comorbid with other psychiatric disorders, even after the veterans return to their civilian lives^1^,^2^,^3^,^4^,^5^.

Numerous neuropsychological studies have reported the presence of cognitive dysfunction associated with PTSD, including memory impairments, attention deficits and learning disabilities^6^,^7^,^8^,^9^,^10^,^11^. In recent years, neuroimaging studies using positron emission tomography (PET) or functional magnetic resonance imaging (fMRI) on PTSD patients have primarily focused on symptom provocation or responses to trauma-related or emotional stimuli. The reviews and meta-analysis of the PTSD related neuroimaging studies suggest that hyperactivation within limbic regions (mainly amygdala and insula) may account for exaggerated fear responses and the persistence of traumatic memories^12^,^13^,^14^,^15^. In addition, it is suggested that hypoactivation of prefrontal regions (mainly anterior cingulate cortex and ventromedial prefrontal cortex) associated with hyperactivation of amygdala may indicate inability of prefrontal regions to inhibit amygdala activity. A recent review article on neuropsychological and neuroimaging studies of PTSD patients highlights the importance of further understanding the PTSD related attentional and inhibitory dysfunctions in order to successfully treat PTSD patients^16^.

In the past decade, functional near infrared spectroscopy (fNIRS), a noninvasive optical imaging method, has been extensively used in the field of neuroimaging for studying functional brain activities. This technique measures the cerebral hemodynamics and oxygenation using near infrared light (670 to 900 nm) which can penetrate through the scalp and skull to reach superficial layers of cerebral cortex, while being partially absorbed by the oxygenated hemoglobin (HbO_2_) and deoxygenated hemoglobin (Hb) in the cerebral blood^17^. In our recent report, we have utilized multi-channel fNIRS to measure prefrontal cortex hemodynamic activations from 16 veterans with PTSD and age-/gender-matched healthy controls during non-trauma-related memory tasks^18^. In particular, the PTSD participants, but not the controls, appeared to suppress prefrontal activity during memory retrieval. This deactivation was more pronounced in the right dorsolateral prefrontal cortex during the retrieval phase. These deactivations in PTSD patients might implicate an active inhibition of dorsolateral prefrontal neural activity during retrieval of working memory. Overall, that study demonstrates that fNIRS could be a portable and complementary neuroimaging tool to study the cognitive dysfunctions associated with PTSD.

In this study, we directed our focus on an objective and quantitative understanding of the selective attention and inhibitory function of cognition in subjects with PTSD, specifically using the color-word matching Stroop task. The Stroop test^19^,^20^ was introduced by J. R. Sroop in^19^ and has been a classic protocol utilized to measure selective attention, cognitive flexibility and processing speed, as well as executive functions. While the color-word Stroop test includes neutral, congruent, and incongruent trials conventionally, researchers often focus on brain activations or performance outcomes during incongruent Stroop trials with respect to either neutral or congruent trials. During an incongruent Stroop test, a word is displayed in an ink color different from what the name of the word means (e.g., word “BLUE” is printed in red ink). Naming the ink color of the word requires more mental effort than just reading the word. The cognitive mechanism of Stroop test is associated with selective attention; the subject has to manage his or her attention by inhibiting or interfering one response in order to promote another. This phenomenon is called ‘Stroop interference’. Numerous functional neuroimaging studies investigating neural correlates of the Stroop interference using PET and fMRI consistently reported activation in lateral prefrontal cortex (LPFC) regions, reflecting the Stroop interference along with activation of the anterior cingulate cortex^21^,^22^. Recent fNIRS studies on the Stroop interference also reported activation in LPFC regions, implicating that LPFC plays a critical role in coping up with the Stroop interference^23^,^24^,^25^,^26^,^27^. Using fNIRS, moreover, Matsuo and colleagues revealed reduced prefrontal activity in a PTSD group when compared to a non-PTSD group during a verbal fluency task^28^.

In this study, fNIRS was used to acquire prefrontal hemodynamic signals from a group of veterans with PTSD and age- and gender-matched healthy controls, in order to investigate both temporal and spatial patterns of prefrontal responses during performance of a Stroop paradigm. The goal of the study was to further illustrate that fNIRS is able to reveal prefrontal activities and deficits in subjects with PTSD during selective attention processes, and that fNIRS could become a portable and complimentary neuroimaging tool to monitor and guide treatments for patients with PTSD.

## Results

### Behavioral measures

Table 1 reports the reaction times (i.e. average time taken to complete a block of 5 Stroop trials) of the control and PTSD groups for Stroop1 (i.e., neutral Stroop trials) and Stroop2 (i.e., incongruent Stroop trials) tasks (See Table 1). For statistical comparison of reaction times, Student t-test was used. As listed in Table 1, each group of healthy and PTSD subjects took significantly longer time to complete Stroop2 task than that to finish Stroop1 task. However, no significant difference in reaction times was observed between the control and PTSD groups for either Stroop1 (p = 0.41) or Stroop2 (p = 0.49) tasks. Both groups responded to Stroop1 and Stroop2 with 100% accuracy.

### Hemodynamic responses

#### HbO_2_ changes induced by Stroop1 and Stroop2 tasks in control group

**Figure 1:**
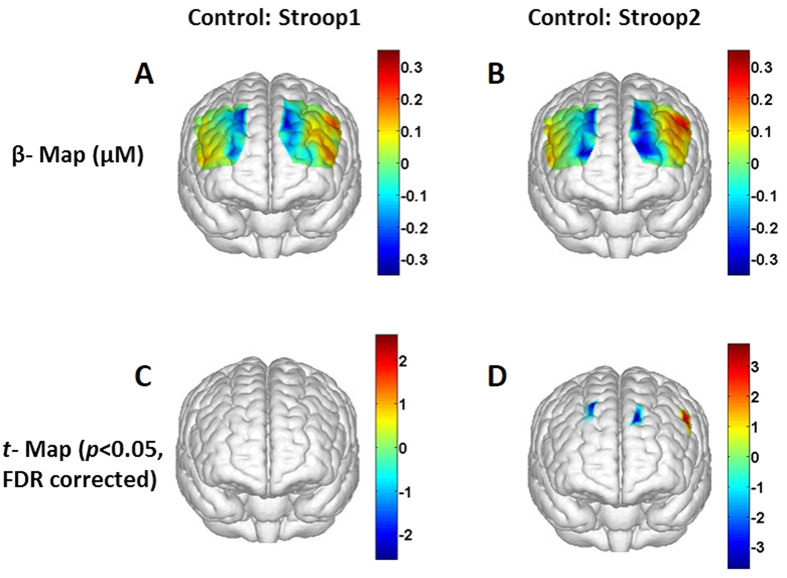
Topographic images of the task-evoked prefrontal activations derived from changes in HbO_2_ in control group. (**A**) Group-averaged prefrontal activation (β-map in μM) evoked by Stroop1 tasks; (**B**) Group-averaged β-map (in μM) evoked by Stroop2 tasks. (**C**) A t-statistical map (t-map) that shows significant activations and/or deactivations (one sample t-test, p < 0.05; FDR corrected) on the brain template during Stroop1 tasks. (**D**) A t-map showing significant activations and/or deactivations evoked by Stroop2 tasks.

For healthy controls, Fig. 1A shows a topographic image of prefrontal HbO_2_ changes (β-map) by Stroop1 task, and Fig. 1C is a 3D rendered t-map (p < 0.05, FDR corrected) to display brain regions where the HbO_2_ changes are significantly different from the baseline readings induced by Stroop1 task. Fig. 1A shows that during Stroop1 task, prefrontal regions towards the midline (line separating two hemispheres) are deactivated and lateral prefrontal regions are slightly activated. Similarly, Fig. 1B illustrates a topographic image of prefrontal HbO_2_ β-map by Stroop2, and Fig. 1D marks regions where the HbO_2_ changes are statistically different from the baseline readings (t-map, p < 0.05, FDR corrected). Close comparison between Fig. 1A,B hinted that the prefrontal β-map induced by Stroop2 exhibits a similar spatial pattern to that evoked by Stroop1 task. Further statistical analysis revealed that Stroop1 task did not stimulate any significant activations and/or deactivations in any channels (Fig. 1C), but Stroop2 task resulted in significant alternations of HbO_2_ in three distinct small regions, as shown in Fig. 1D.

**Figure 2:**
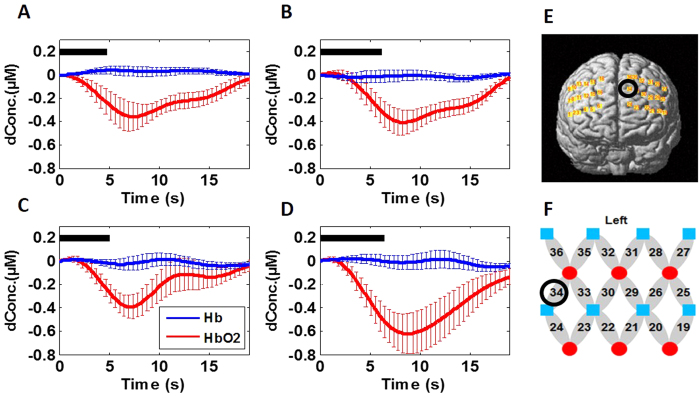
Mean Stroop-evoked hemodynamic changes of HbO2 in left DLPFC (i.e., channel 34 shown in Panels (**E**,**F**)) from both groups. (**A**,**B**) Group-averaged HbO_2_ response from the healthy group evoked by Stroop1 and Stroop2 tasks, respectively. (**C**,**D**) Group-averaged HbO_2_ response from the subjects with PTSD evoked by Stroop1 and Stroop2, respectively. In all four panels, the thick black bars represent the stimulation periods; the red lines represent the mean time courses of HbO_2_; the blue lines represent the mean time courses of Hb; the error bars represent the standard errors of mean. (**E**) It shows the position or location of channel 34 on a standard human brain template. (**F**) It shows the configuration of fNIRS channels on the left frontal cortex. Red circles represent light sources, blue squares represent detectors, and gray ellipses represent the nearest source-detector pairs (as channels) to measure the brain activities.

To be more specific for demonstrating deactivations and/or activations at the identified regions, we plot group-averaged HbO_2_ time courses evoked by Stroop1 and Stroop2 at channel 34 around the medial LPFC (see Fig. 2E,F for channel location), as shown in Fig. 2A,B, respectively. These two figures show clear reduction of HbO_2_ responses in the cortical region near Channel 34 by both Stroop trials (i.e., neutral versus incongruent trails) with very similar magnitude and temporal pattern, implying that no significant difference existed between HbO_2_ responses by the two Stroop trials at this cortical region.

**Figure 3:**
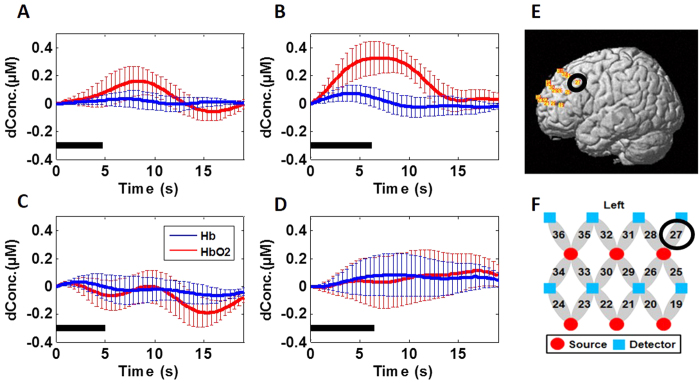
Mean Stroop-evoked hemodynamic changes of HbO_2_ in left pars opercularis (see channel 27 shown in Panels **E**,**F**) from both groups. (**A**,**B**) Group-averaged HbO_2_ response from the healthy group evoked by Stroop1 and Stroop2 tasks, respectively. (**C**,**D**) Group-averaged HbO_2_ response from the subjects with PTSD evoked by Stroop1 and Stroop2, respectively. In all four panels, the thick black bars represent the stimulation periods; the red lines represent the mean time courses of HbO_2_; the blue lines represent the mean time courses of Hb; the error bars represent the standard errors of mean. (**E**) It shows the position or location of channel 27 on a standard human brain template. (**F**) It shows the configuration of fNIRS channels on the left frontal cortex. Red circles represent light sources, blue squares represent detectors, and gray ellipses represent the nearest source-detector pairs (as channels) to measure the brain activities.

On the other hand, channel-wise data analysis revealed a significant increase in HbO_2_ response (p = 0.007) near left LPFC [Brodmann Area (BA) 44, Channel 27; see Fig. 3E,F for channel location]. Then, group-averaged HbO_2_ time courses were taken and plotted from channel 27 under both Stroop1 and Stroop2 tasks, as Fig. 3A,B show, respectively. Close visual inspection between these two figures suggests that Stroop2 evoked more HbO_2_ signals than Stroop1, resulting in significant changes with respect to the baseline readings (as seen in Fig. 1D).

### HbO_2_ changes induced by Stroop1 and Stroop2 tasks in PTSD group

**Figure 4:**
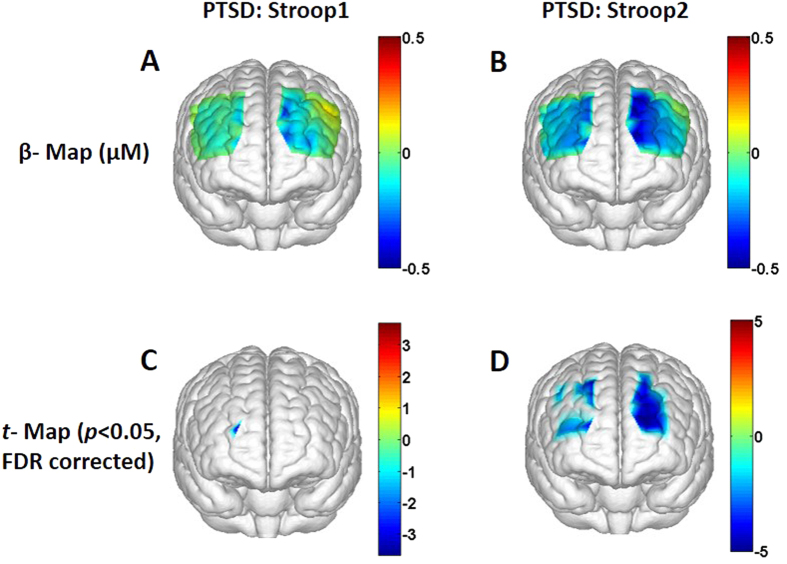
Topographic images of the task-evoked prefrontal activations derived from changes in HbO_2_ from the PTSD group. (**A**) Group-averaged prefrontal activation (β-map in μM) evoked by Stroop1 tasks; (**B**) Group-averaged β-map (in μM) evoked by Stroop2 tasks. (**C**) A t-statistical map (t-map) that shows significant activations and/or deactivations (one sample t-test, p < 0.05; FDR corrected) on the brain template during Stroop1 tasks. (**D**) A t-map showing significant activations and/or deactivations evoked by Stroop2 tasks.

For the PTSD group, Fig. 4A shows a topographic image of prefrontal HbO_2_ β-map by Stroop1 task, and Fig. 4C is a 3D rendered t-map (p < 0.05, FDR corrected) to mark regions that have significant HbO_2_ changes from the baseline induced by Stroop1. Fig. 4A shows that prefrontal regions are mildly deactivated by Stroop1 task. Similarly, Fig. 4B illustrates a topographic image of prefrontal HbO_2_ β-map by Stroop2. It is clear that deactivations induced by Stroop2 task are widespread across the prefrontal cortex. Fig. 4D marks regions where the HbO_2_ changes are statistically different from the baseline readings (t-map, p < 0.05, FDR corrected). Overall, in the PTSD group, spatial extent of prefrontal deactivation induced by Stroop2 task has been observed much larger than that induced by Stroop1 task.

To better understand and demonstrate deactivations and/or activations at the identified regions in the PTSD group, we also plot group-averaged HbO_2_ time courses evoked by Stroop1 and Stroop2 at channel 34 (as we did for the control group in Fig. 2A,B), as shown in Fig. 2C,D, respectively. These two figures also show clear reduction of HbO_2_ responses near the medial LPFC by both Stroop trials, with a larger deactivation magnitude by Stroop2 task. Regarding HbO_2_ activation, for easy and quantitative comparison between the control and PTSD groups, we also plot group-averaged HbO_2_ time courses from channel 27 under both Stroop tasks, as Fig. 3C,D show, respectively. Close visual inspection between these two figures suggests that neither of the Stroop tasks evoked much HbO_2_ signals with respect to the baseline readings (as implied in Fig. 4D).

It is clear that deoxy-hemoglobin responses in both control and PTSD groups under both Stroop tasks were relatively low and insignificant (See Figs 2A–D and 3A–D). Therefore, Hb responses were excluded from further data analysis.

#### HbO_2_ changes induced by Stroop interference in control versus PTSD group

**Figure 5:**
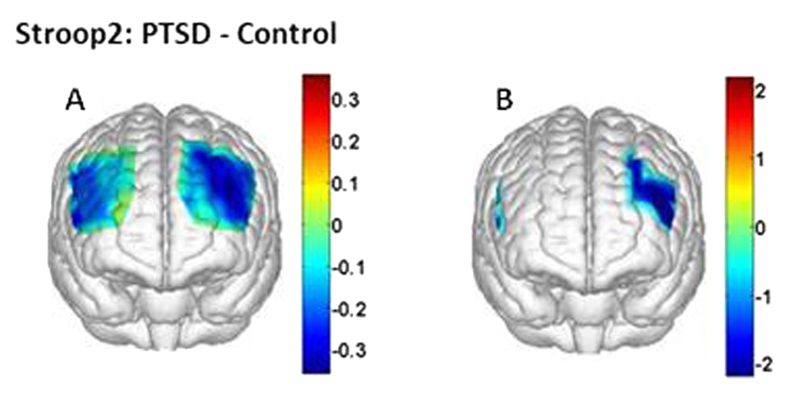
Topographic images of the prefrontal activations derived from HbO_2_, showing differences between the PTSD and control groups in response to Stroop2 (incongruent Stroop) tasks. (**A**) Group-averaged differences in activations or β-maps (in μM) between the two groups. (**B**) A t-map (p-value < 0.1; FDR corrected) showing the regions of significantly different activations and deactivations between the groups.

**Figure 6:**
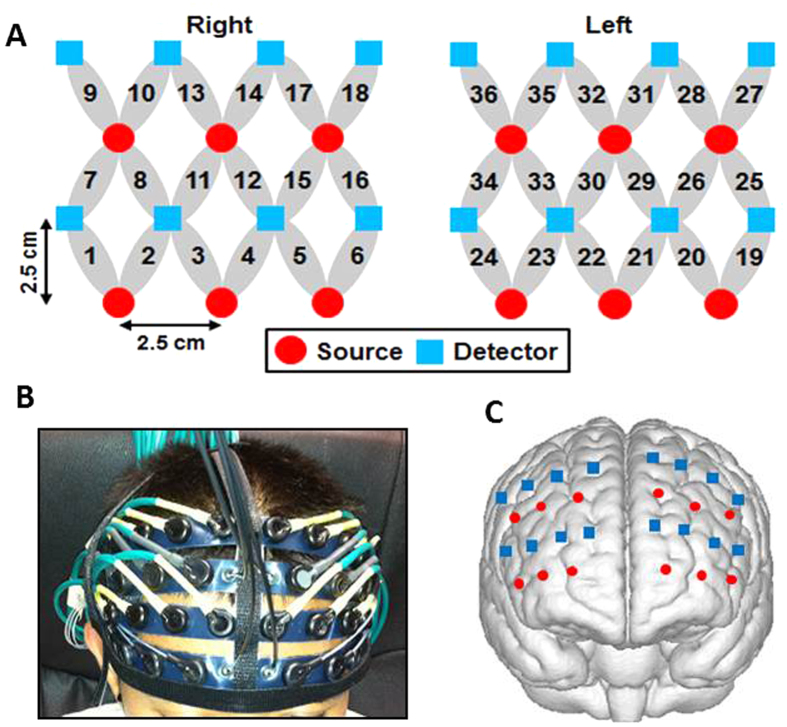
Configuration and cortical position of the multi-channel fNIRS probe array. (**A**) Configuration of the fNIRS probe. Red circles represent light sources, blue squares represent detectors, and gray ellipses represent the nearest source-detector pairs (as channels) to measure the brain activities. (**B**) Placement of the fNIRS probe on a participant’s forehead. (**C**) Co-registration of the sources and detectors on a standard brain atlas template. The probe partially covers the frontopolar, dorsolateral, and ventrolateral prefrontal regions on both hemispheres. The anatomical position of each channel on the brain atlas is detailed in Table 3.

The main focus of this study was to explore differences of prefrontal hemodynamic signals in response to Stroop interference between a group of veterans with PTSD and age- and gender-matched healthy controls, in order to investigate possible deficits of selective attention processes in PTSD subjects. Furthermore, both Figs 1C and 4C do not show any significant cortical activation or deactivation evoked by Stroop1 task. Thus, we directed our attention to only Stroop2 task hereafter. Specifically, we found distinct differences of HbO_2_ changes in both temporal and spatial patterns between the two subject groups only during incongruent trials. Figure 5A shows a differential topographic HbO_2_ β-map between the PTSD and control groups, while Fig. 5B marks regions where the differential HbO_2_ responses between the two groups are significantly different (t-map, p < 0.1, FDR corrected) in response to incongruent Stroop trials. Specifically, channel-wise data analysis revealed that nine out of total 36 channels exhibited significantly reduced HbO_2_ values in the PTSD group when compared to the control group. Two channels were located in right pars triangularis [BRODMANN AREA45, Channels 1 (p = 0.04) and 7 (p = 0.04)]. Seven channels were located on the left LPFC: three on the left pars triangularis [BA 45, Channels 19 (p = 0.02), 25 (p = 0.03) and 26 (p = 0.02)] and four on the left dorsal LPFC (DLPFC) [BAs 9 & 46, Channels 20 (p = 0.03), 29 (p = 0.02), 30 (p = 0.04), 32 (p = 0.03)]. For better visualization of the mentioned channel locations, Fig. 6 presents the configuration and cortical position of the multi-channel fNIRS probe array.

#### Correlation between behavioral and hemodynamic measures

Linear regression analyses were performed between the task reaction times and corresponding HbO_2_ response magnitude (i.e., β-values) at each channel during Stroop1 and Stroop2 tasks. For Stroop1 task, neither control nor PTSD group showed any significant correlation between the task reaction times and HbO_2_ responses in any of the 36 channels. However, for Stroop2 task, two of total 36 channels exhibited a significant negative correlation between the task reaction times and HbO_2_ responses from only the PTSD group, Those channels were located in left DLPFC [BA 9, Channels 28 (R = −0.55, p = 0.03) and 31 (R = −0.52, p = 0.04)]. The control group did not present a Stroop2-activated liniear correlation between the task reaction times and HbO_2_ responses.

#### Left DLPFC as Region of Interest (ROI)

While the above t-maps shown in Figs 1D, 4D and 5B were corrected by the FDR (false discovery rate), the correction was less stringent for Type I errors compared to the familywise error correction. In particular, the FDR-corrected confidence level for Fig. 5B was set to be 90% with a p value less than 0.1. Therefore, it was necessary to perform an ROI-based data analysis according to recently published and accepted findings^23^,^24^,^25^,^26^,^27^, which consistently reported bilateral LPFC activation with predominant activation in left LPFC due to Stroop interference^23^,^24^.

**Figure 7:**
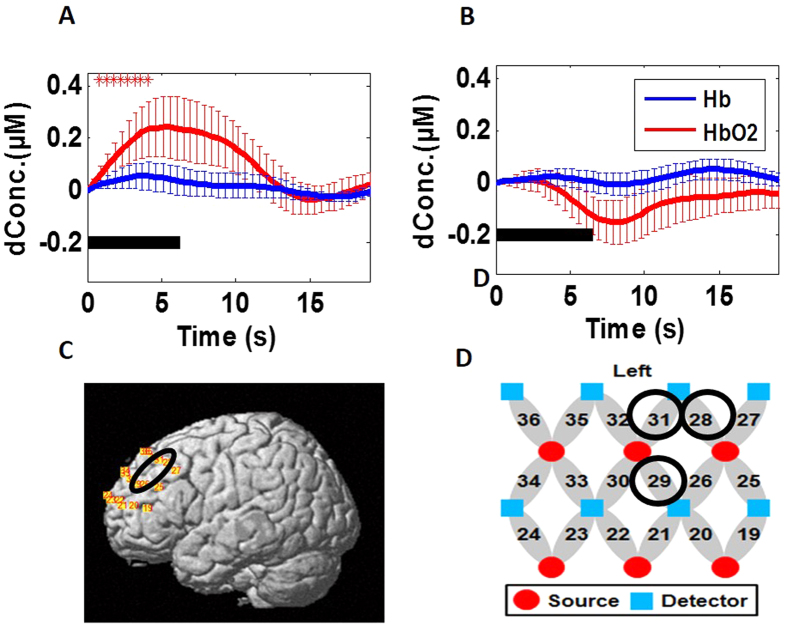
Mean Stroop2-evoked hemodynamic responses from the selected ROI, which includes channels 28, 29 and 31 from left DLPFC, from both groups. (**A**) Group-averaged, ROI-averaged HbO_2_ responses from the control group evoked by Stroop2 tasks. (**B**) Group-averaged, ROI-averaged HbO_2_ responses from the PTSD group evoked by Stroop2 tasks. In both panels, the thick black lines represent the stimulation period; the thick red lines represent the mean time courses of changes in HbO_2_; the thick blue lines represent the mean time courses of changes in Hb; the error bars represent standard errors of mean. Asterisks ‘*’ indicate significant (p < 0.05) HbO_2_ responses with respect to the mean baseline at different time points.

**Figure 8:**
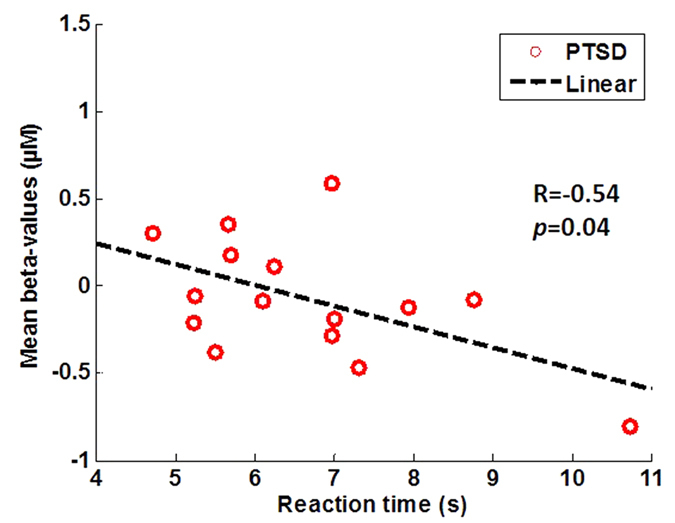
Correlation between individual task reaction times and ROI-averaged HbO_2_ responses (i.e., β-values) from the ROI (i.e., channels 28, 29 and 31) in the left DLPFC under Stroop2 task, taken from PTSD subjects.

In this current study, we thus selected Channels 28, 29 and 31 (See Fig. 7C,D for channel locations) as the ROI, which corresponded to the left DLPFC and roughly matched the activation sites in aforementioned studies. Given this ROI, we investigated the ROI-averaged HbO_2_ signals induced by the incongruent Stroop task in both control and PTSD groups. Fig. 7A,B plot group-averaged, ROI-based temporal HbO_2_ responses from the control and PTSD group, respectively. The control group shows a Stroop2-evoked significant increase (p < 0.05) in HbO_2_ response during the first several seconds of stimulation from the mean baseline within the ROI (See Fig. 7A), but the PTSD group did not show any significant difference in HbO_2_ response from the mean ROI-averaged baseline (See Fig. 7B). Furthermore, Stroop2 task resulted in a marginally significant negative correlation (R = −0.54, p = 0.04) between the individual task reaction times and the ROI-averaged HbO_2_ responses from the PTSD group (See Fig. 8) while no significant correlation was seen in the control group.

## Discussion

### Experimental Observations

The present study compared and contrasted prefrontal hemodynamic responses during the Stroop color-word neutral and incongruent tasks between the veterans with PTSD and age- and gender- matched controls. The observed results under the incongruent task are meaningful and thus summarized first as follows:

Observation 1: When being compared to the baseline, the healthy group exhibited significant hemodynamic activation in the left pars opercularis of control subjects (see Figs 1D and 3B).

Observation 2: When being compared to the baseline, both the healthy and PTSD groups showed significant hemodynamic deactivations, particularly in the bilateral frontal polar area (FPA) and bilateral DLPFC regions towards the midline (see Figs 1B,D and 4B,D).

Observation 3: The PTSD group presented reduced hemodynamic activity in the left DLPFC and bilateral pars triangularis (BA 45) significantly when being compared to the control group (see Fig. 5).

Observation 4: The ROI-based analysis confirmed that healthy subjects displayed significant hemodynamic activation in the selected ROI (i.e., left DLPFC) (see Fig. 7A), while the PTSD group did not show any significant hemodynamic activation or deactivation in the ROI during the same Stroop task (see Fig. 7B).

Observation 5: Regarding the brain imaging and behavioral relationship, we observed a marginally significant negative correlation between the task reaction time and HbO_2_ response amplitude taken from the ROI of the PTSD group (see Fig. 8).

### Interpretations

#### Interpretation for Observation 1: Activation of left pars opercularis by Stroop2 task in control subjects

Hemodynamic activations observed in our study in the LPFC regions, especially in the left DLPFC, were consistent with numerous fNIRS and fMRI studies that reported earlier neural correlates of Stroop interference^23^,^24^,^25^,^26^,^27^,^29^. Particularly, a recent fNIRS study has demonstrated that Stroop interference evoked significantly the left DLPFC after acute bout of exercise, and that such increased activation in the mentioned cortical region in turn matched with improved Stroop task performance after the exercise^23^. Therefore, it is reasonable to expect that cerebral hemodynamic activation in the left DLPFC is highly associated or linked with Stroop interference.

#### Interpretation for Observation 2: Deactivations in the bilateral FPA and bilateral DLPFC in both groups

In our study, we observed deactivations in channels from the DLPFC (BA 9) area towards midline and FPA (BA 10) during Stroop2 task in healthy subjects. However, the deactivations observed were not statistically significant compared to the baseline for most of channels (see Fig. 1D). For the PTSD group, more channels from similar DLPFC area towards midline and FPA illustrated deactivations (see Fig. 4D) than the healthy group. In comparison, functional NIRS studies by Matsuda and colleagues reported deactivations in dorsal prefrontal cortex (DPFC, BAs 9 and 10) of adults and children during videogame tasks^30^,^31^. These fNIRS studies implicated that deactivations observed in DPFC were due to neural inhibition derived from continuous attention demand for video game tasks. A meta-analysis of 9 PET studies also reported regional cerebral blood flow (rCBF) decreases in medial frontal regions running along a dorsal-ventral axis (BAs 8, 9, 10 and 32), during 9 goal-directed tasks (such as spatial attention, visual search, and language tasks)^32^. The goal-directed tasks, which were analyzed in the meta-analysis report, had nothing in common except that all tasks required a response to certain visual stimuli. In an fMRI study, Mazoyer and colleagues demonstrated deactivations in dorsal medial prefrontal cortex (DMPFC; BAs 9, 10) during sustained attention to visual stimuli. In the meantime, a negative correlation between the load of sustained attention towards visual stimuli and blood oxygenation level dependent (BOLD) signal in DMPFC was reported as well^33^. Based on those reported findings, therefore, it is reasonable to attribute the deactivations observed in our study as the response to visual stimuli or sustained attention to visual stimuli rather than Stroop specific deactivations.

#### Interpretation for Observations 3 and 4: Reduced response in left DLPFC in PTSD group than healthy group

For between-group differences in behavioral measures, we did not observe any significant difference in either performance accuracy or reaction times during Stroop interference task between the control and PTSD groups. However, in hemodynamic measures, during Stroop2 task, PTSD subjects showed significant reduction in HbO_2_ responses in right pars triangularis (BA 45, Channels 1 and 7), left pars triangularis (BA 45, Channels 19, 25 and 26) and left DLPFC (BAs 9 & 46, Channels 20, 29, 30, 32) when compared to controls (see Fig. 5). This reduced response was expected since the control group exhibited significant activation in left lateral prefrontal regions, especially in the left DLPFC region to overcome Stroop interference, whereas the PTSD group failed to activate left lateral prefrontal regions. This result might implicate the difficulty experienced by PTSD subjects in coping up with Stroop interference. Therefore, restoration of left DLPFC activity in PTSD participants might improve their selective attention performance. Such improvements can be monitored and/or revealed by multi-channel fNIRS combined with the conventional Stroop color-word task.

The Stroop color-word test used in this study can also be thought as a measure of inhibitory control. Each participant performing this task had to inhibit the automatic or prepotent response of word naming in order to choose the appropriate color name of the word. During the Stroop2 color-word matching task, we observed significant reduction/deactivation in HbO_2_ responses in bilateral pars triangularis (BA 45) in the PTSD group than in the control group. Pars triangularis is a part of ventrolateral prefrontal cortex (VLPFC). This reduction in VLPFC activations during Stroop2 task may suggest abnormal inhibitory mechanism in PTSD subjects. A recent fMRI study reported that during the task involving inhibitory control (such as go-nogo task), PTSD patients exhibited reduced activation in the inferior frontal cortex, VLPFC and DLPFC relative to controls^34^.

#### Observation 5: Negative correlation between the brain imaging and behavior

Furthermore, our results illustrated that the brain responses within the selected ROI during Stroop2 task were negatively correlated with the reaction time (see Fig. 8) in the PTSD group. This observation may imply that the attenuated activations may be well correlated with inhibitory control. Several neuroimaging studies investigating neural correlates of emotion regulation have reported increased PFC activity and associated decrease in amygdala activity during successful emotion regulation^35^,^36^,^37^,^38^,^39^. These published studies suggest that activities in several sub-regions of PFC, such as VLPFC and DLPFC, are involved in emotion control, including inhibition of Stroop interference. The reason for not being able to observe such a negative correlation in the control group could be attributed to the relative easiness of the tasks for the healthy subjects.

### Limitation

A few limitations exist in this current study, including both the protocol design and technical limitations. In protocol design, Stroop1 task (i.e., neutral task) was too easy for the study, based on two experimental outcomes: (1) the brain responses to Stroop1 did not show any significant difference from the baseline readings, in both healthy and PTSD groups (see Figs 1C and 4C); (2) there was no significant difference in brain response to Stroop1 between the healthy and PTSD group (see Fig. 2A vs 2C; Fig. 3A vs. 3C). Similarly, in behavioral measures, both groups took longer time to complete Stroop2 task when compared to Stroop1 task, as expected because of the Stroop interference effect. However, no significant difference in reaction times and accuracy was observed between the two groups (see Table 1). It is clear that more challenging or complex neural and interference Stroop tasks should be designed or used in future studies in order to gain better contrasts in brain responses between neutral/congruent versus incongruent tasks and between healthy and PTSD subjects.

Furthermore, there are three technical limitations in the present study, similar to what we discussed in our previous work^18^. First, we had only a limited region of the prefrontal cortex covered by the fNIRS probe, so functions of other cortical regions involved during the Stroop tasks were not studied. Second, most veterans with PTSD in the study suffered from comorbid conditions, which could confound or bias our measured signals that were considered only from PTSD conditions. Lastly, it was possible that our fNIRS signals used for cerebral-hemodynamic quantification could include some contamination from extra cranial vasculature, such as from the scalp and skull. This contamination may be minimized by regression of the signals from superficial layers that are recorded with short source-detector separations of 0.8–1 cm.

## Conclusions

In the present study, functional near infrared spectroscopy was utilized to assess the involvement of the prefrontal cortex in selective attention processes among 15 veterans with PTSD and 13 age-/gender- matched healthy controls. While performing Stroop (color-word) incongruent task, healthy controls showed hemodynamic activations in lateral prefrontal cortex regions, especially in left DLPFC and left Pars opercularis, whereas veterans with PTSD failed to activate those cortical regions during the same Stroop task. These observations might implicate difficulty experienced by the PTSD subjects in coping up with Stroop interference. In addition, significant negative correlation was observed between task reaction times and HbO_2_ responses from left DLPFC during color-word matching task. The present study clearly demonstrates that fNIRS is a portable and complimentary neuroimaging tool to study the neural correlates of selective attention and interference in subjects with PTSD.

## Material and Methods

### Participants

A Total of 15 combat exposed veterans diagnosed with PTSD (all males, right-handed, mean ± SD age  = 29.1 ± 9.0 years) were recruited in this study. The comorbid conditions diagnosed in these 15 veterans are provided in Table 2. The comorbid conditions diagnosed are attention deficit hyperactivity disorder (ADHD) (n = 4), major depressive disorder (n = 5), alcohol dependence (n = 4), musculoskeletal pain (n = 4), insomnia (n = 3), history of blast exposure (n = 1), anxiety disorder (n = 2), mild traumatic brain injury (mTBI) (n = 3), and learning disorder (n = 2). The hemodynamic activations in veterans with PTSD were compared against 13 age- and gender- matched healthy controls (all males, right-handed, mean ± SD age  = 33.3 ± 10.3 years). The protocol used in the study was reviewed and approved by the Institutional Review Board (IRB) of the University of Texas at Arlington (UTA). The methods were carried out in accordance with approved guidelines by IRB of UTA. Written informed consent was obtained from all participants prior to the fNIRS scan.

### Tasks and paradigm

**Figure 9:**
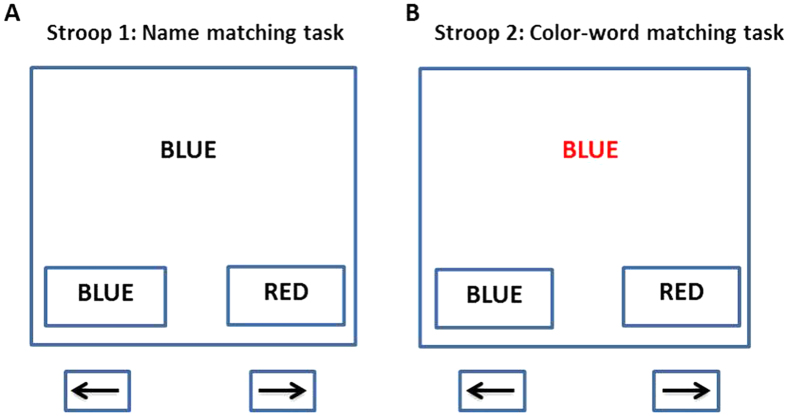
Examples of Stroop trials used in this study. (**A**) Stroop1 (i.e., neutral Stroop task) trial: Participants should match the word name given at the center of the screen to word name given in options at bottom of the screen, by pressing the appropriate left or right arrow key. (**B**) Stroop2 (i.e., incongruent Stroop task) trial: Participants should match the ink color of the word given at the center of the screen to word name given in options at the bottom of the screen, by pressing the appropriate left or right arrow key.

The paradigm used in this study consisted of two sessions. The first session, named Stroop1, was a similar version of neutral trails of the Stroop color-word task. It consisted of word-name matching tasks and was a simple paradigm where participants had to match the name of the word displayed at the center of a computer screen to either one of the options displayed at the bottom of the screen, by using arrow buttons on the keyboard (See Fig. 9A). In this task, all of the words were written in black ink color. The second session was called Stroop2 and made of incongruent trails of the Stroop color-word task. During this session, participants had to match the ink color of the displayed word, whose color was different from the name of the word itself, to the name of the word given in options (See Fig. 9B).

All the participants were asked to sit comfortably before a computer, and complete a session of Stroop1 task, followed by a session of Stroop2 task, while their brains were scanned by multichannel fNIRS. Within each Stroop1 and Stroop2 session, the paradigm consisted of a baseline (resting) period of 30 sec, followed by eight blocks of stimulation-and-resting sequences. Within each block, five random Stroop trials (generated by computer) were given to the subjects. The subjects were instructed to select the appropriate option accurately without any time constraint. Between two blocks, there was an inter-stimulus or resting interval of 15 sec. Before each fNIRS measurement session started, all participants were trained to practice a few trials of Stroop1 and Stroop2 tasks. An experimenter observed the course of practice to confirm that the participants understood the paradigm correctly. The accuracy of each participant’s performance in each of the Stroop task was measured by the percentage of correctly performed trials divided by the total number of trials ( =5 trials × 8 blocks) in each session. The reaction time measured for each participant was defined as time taken to complete a single block of five Stroop trials.

### Functional near infrared spectroscopy

#### Instrument

A multi-channel, continuous wave, fNIRS imaging system (Cephalogics LLC., Boston, MA) was used to acquire each participant’s prefrontal hemodynamic activities during performance of the tasks^40^. The system consisted of near infrared light sources (light emitting diodes, LEDs) emitting at two wavelengths (750 nm and 850 nm) of light and avalanche photodiodes (APDs) as detectors. This system provided fNIRS signals with a sampling rate of 10.8 Hz. For this specific experiment, the fNIRS probe array composed of 12 pairs of light sources and 16 detectors, was placed symmetrically over both hemispheres of the participant’s forehead (see Fig. 6). The bottom row of 6 pairs of light sources in the probe was placed just above the participant’s eyebrows, and its midpoint was ~3.5 cm above the nasion. This probe provided a total of 36 measurements (channels) when only the nearest source-detector pairs were considered (the nearest source-detector separation was 2.8 cm). Other measurements from larger source-detector separations were excluded because their signals were too weak to be scientifically meaningful. The probe assembly was constructed with low-weight optical fibers (TechEn Inc., Boston, MA) and thin polyethylene film to ensure participants’ comfort during the experiment.

#### Spatial registration

To estimate the cortical regions covered by the fNIRS probe, a spatial registration procedure was performed among six randomly selected participants^41^. Once the fNIRS probe was placed on the subject’s forehead (see Fig. 6B), the positions of light sources and detectors along with five cranial landmarks (the nasion, inion, left and right pre-auricular points, and vertex) were measured using a PATRIOT motion tracking system (Polhemus, Colchester, Vermont, USA.). The cranial landmarks served as mediators to convert the real-world stereotaxic coordinates of the optodes to the Montreal Neurological Institute (MNI) coordinates used in a standard brain MRI atlas based on the affine transformation^42^. Figure 6C shows the registered optode positions (averaged over six participants) on the standard human brain atlas. The probe partially covered the frontopolar area (BA 10), dorsolateral prefrontal cortex (DLPFC; BAs 9 and 46), pars triangularis (BA 45) and pars opercularis (BA 44). A detailed report is given in Table 3. Because the fNIRS probe was carefully placed on each individual participant’s forehead by referring to the nasion and eyebrows, the registered optode positions across individual participants were relatively consistent (positional variations were about 5 mm) in comparison with the separation of two neighboring measurement channels.

#### Data screening and processing

The temporal evolutions of light intensities measured during both Stroop1 and Stroop2 sessions were screened and processed using a publically available toolbox (Homer, http://www.nmr.mgh.harvard.edu/PMI/resources/ homer/home.htm )^43^. First, the raw light intensity signals were visually inspected to exclude blocks associated with significant motion artifacts (with an intensity fluctuation of 15% or larger from the baseline) during each session. Then the resulted signals free from motion artifacts were low-pass filtered at a cut-off frequency of 0.2 Hz to remove electronic noise and systemic noise (cardiac and respiratory oscillations) and high-pass filtered at a cut-off frequency of 0.01 Hz to remove any possible slow baseline drift. Then, changes of oxygenated and deoxygenated hemoglobin concentrations (i.e., ΔHbO_2_ and ΔHb, respectively) relative to the baseline were quantified following the modified Beer-Lambert Law^44^. At this step, we estimated the differential pathlength factor (DPF) to be 6.2 at 750 nm and 5.8 at 850 nm, based on published data for adult heads^45^.

### Linear regression for reaction times and HbO_2_ changes

For both Stroop tasks, correlations between the participants’ reaction times and corresponding HbO_2_ changes (i.e., β-values) were tested using linear regression at each measurement channel for both control and PTSD groups, separately. In addition, correlations between participants’ reaction times and ROI-averaged HbO_2_ responses (i.e., Mean β-values over the ROI) were calculated for both the control and PTSD groups, separately.

#### General linear model (GLM) analysis

To quantify cerebral hemodynamic activities during Stroop1 and Stroop2 tasks, a model-based statistical analysis tool, general linear model (GLM), was utilized. GLM analysis has been increasingly utilized to analyze fNIRS data over the last decade to identify cortical areas which are significantly stimulated during a given task^46^,^47^,^48^,^49^. In GLM, a hemodynamic response function (HRF) is used to serve as a model to predict the change in HbO_2_ signals due to task stimulation; GLM can be expressed by equation (1):


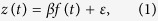



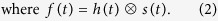


In equation (1), *z(t)* represents the temporal profile of HbO_2_ or Hb changes at each measurement channel, *f(t)* is the predicted stimulation-specific response and is expected to match the profiles of measured signals, *h(t)* is a given HRF, and *s(t)* is the stimulation-specific boxcar function for a given task. Moreover, *β* is the estimated amplitude of ΔHbO_2_, while ε is an error term to account for any residual due to the mismatch between the actual data and the model. By fitting equation (1) to the temporal profile of ΔHbO_2_ obtained from each channel from each participant, we would be able to obtain (i) the estimated amplitude, (ii) its variance, and thus (iii) a statistical t-value representing the statistical significance of the brain activation at each respective channel.

Ideally, the HRF derived from the fNIRS signals via an event-related experimental paradigm would be appropriate for this study. However, to the best of our knowledge, such an HRF is not available for attention-evoked responses in the prefrontal cortex. Therefore, we used a standard HRF derived from BOLD fMRI as a surrogate^50^. By fitting the predicted stimulation response function to the channel-wise, temporal profiles of HbO_2_ responses, the amplitudes (expressed by *β*-values in μM) of prefrontal activations or deactivations in response to each Stroop task were obtained^48^.

#### Random effects

For group-level hemodynamic measures, *β*-values at each channel were calculated by averaging *β*-values across each group of the subjects. Random-effect analysis was performed in order to generate statistically meaningful quantities at the group level. This was accomplished by conducting the one- sample t-test on *β*-values from all subjects at each channel for each subject group. For both control and PTSD subjects, group-level t-statistic analysis parameters (expressed by t-values) were obtained to show statistically increased and/or decreased brain activations during Stroop1 and Stroop2 tasks when compared to the baseline readings. To identify the regions showing significant differences in brain activations or deactivations between the control and PTSD groups, two sample t-tests were performed on *β*-values obtained from individual subjects. Both t-statistic values (expressed by t-values) and p-values were derived from the t-tests for each channel and used to generate t-maps in topographic images.

#### Topography

Topographic images of prefrontal activations and/or deactivations were generated using EasyTopo, an optical topography toolbox developed in our lab^42^. EasyTopo overlays 2D images of HbO_2_ or Hb activations/deactivations over a standard brain MRI atlas after 2D angular interpolation of the channel-wise activation data in a spherical coordinate system. In this study, the channel-wise β-values derived from GLM analysis and t-values from subsequent statistical comparisons were interpolated to generate activation maps (β-maps and t-maps) induced by Stroop1 and Stroop2 tasks.

## Additional Information

**How to cite this article**: Yennu, A. *et al.* Prefrontal responses to Stroop tasks in subjects with post-traumatic stress disorder assessed by functional near infrared spectroscopy. *Sci. Rep.* **6**, 30157; doi: 10.1038/srep30157 (2016).

## Figures and Tables

**Table 1:** Task-dependent reaction times (sec) of control and PTSD groups.

	Control group		PTSD group	
	Stroop 1	Stroop 2	Stroop 1	Stroop 2
Reaction time (Mean ± SD; sec)	4.78 ± 0.62	6.28 ± 0.74	5.06 ± 1.12	6.54 ± 1.27
*p* value within each group	<10^−6^		<10^−4^	
*p* value between two groups	>0.41	>0.49		

**Table 2:** Comorbid conditions of veteran participants with PTSD.

Participant number	Comorbidities
1	PTSD and Anxiety Disorder (Not otherwise specified)
2	PTSD, ADHD and Substance Abuse Disorder
3	PTSD, ADHD and Musculo-skeletal pain
4	PTSD, Anxiety Disorder (Not Otherwise specified), Musculo-skeletal pain and insomnia
5	PTSD, Musculo-skeletal pain and Insomnia
6	PTSD, Alcohol Dependence, History of blast exposure and learning disorder
7	PTSD, Major depressive episodes and Alcohol Abuse
8	PTSD, Major depressive episodes, Insomnia and Musculo-skeletal pain
9	PTSD and Moderate Traumatic brain injury
10	PTSD, Major depressive episodes, Moderate TBI and History of learning disorder
11	PTSD, Major depressive episodes, ADHD and Insomnia
12	PTSD, Depression symptoms, Attentionality symptoms,
13	PTSD, Moderate TBI, Major depressive episodes, Alcohol abuse
14	PTSD, ADHD and Alcohol abuse
15	PTSD

**Table 3:** Registration of the fNIRS channel positions on a standard brain atlas.

Hemisphere	Brodmann Area (BA)	Channel #
Right	BA9	13, 14, 15, 16, 17, 18
	BA10	4, 5, 6
	BA44	9, 10
	BA45	1, 7, 8
	BA46	2, 3, 11, 12
Left	BA9	28, 31, 32, 33, 34, 35, 36
	BA10	22, 23, 24
	BA44	27
	BA45	19, 25, 26
	BA46	20, 21, 29, 30

The channel numbers are defined in Fig. 6A.
